# The Double Burden: Climate Change Challenges for Health Systems

**DOI:** 10.1177/11786302241298789

**Published:** 2024-11-20

**Authors:** Flavio Pinheiro Martins, Marco Antonio Catussi Paschoalotto, Jose Closs, Meike Bukowski, Mariana M Veras

**Affiliations:** 1 The Bartlett School of Sustainable Construction, UCL—University College London, London, UK; 2 Research Center in Political Science (CICP), School of Economics, Management and Political Science, University of Minho, Braga, Portugal; 3 UNU-EGOV, United Nations University Operating Unit on Policy-Driven Electronic Governance, Guimarães, Portugal; 4 Laboratory of Environmental and Experimental Pathology—Hospital das Clínicas, Faculty of Medicine, University of São Paulo, Sao Paulo, Brazil; 5 Department of Geography and Geology, University of Salzburg, Salzburg, Austria

**Keywords:** Climate change, Health systems, Collaboration, Public health infrastructure, Climate Health

## Abstract

Climate change presents significant challenges to human health and health systems, and there is a critical need for health systems to adapt and become more resilient in order to effectively mediate the impacts of climate change on population health. This paper analyzes existing academic literature to identify key themes, trends, and research gaps at the intersection of climate change and health systems. Utilizing a scoping review of 179 studies, we explore how health systems can enhance their resilience through effective governance, sustainable financing, resource generation, and adaptive service delivery. Our findings emphasize the importance of integrating climate considerations into health system governance, mobilizing innovative financial resources, and adapting infrastructure and workforce capacities to address climate-related health challenges. The study highlights the need for continued interdisciplinary research and targeted interventions to ensure health systems are equipped to promote equity and protect vulnerable populations in the face of climate change. These insights contribute to the development of climate-resilient health systems and identify crucial areas for future research.

## Introduction

Climate change, as defined by the Intergovernmental Panel on Climate Change (IPCC) in its Sixth Assessment Report, refers to “a change in the state of the climate that can be identified (eg, by using statistical tests) by changes in the mean and/or the variability of its properties, and that persists for an extended period, typically decades or longer.”^1^ This change is primarily caused by human activities, particularly the emission of greenhouse gases from burning fossil fuels. Climate change affects human health through increased frequency and intensity of extreme weather events, air pollution, and waterborne illnesses. With the influx of studies in the post-COVID era,^2^ there is now sufficient knowledge to examine the intersection between climate change and health. However, further research is needed, especially regarding vulnerable populations.^3^

These health impacts of climate change pose significant challenges to health systems worldwide. Health systems consist of all organizations, people, and actions whose primary intent is to promote, restore or maintain health. They aim to improve population health outcomes in an equitable way without overburdening people with health care costs, working towards the goal of universal health coverage (UHC).^4,5^ However, climate change threatens this objective in multiple ways. It increases health risks through more frequent and severe extreme weather events, disrupts food systems, and exacerbates both infectious and noncommunicable diseases.^4^ These impacts disproportionately affect vulnerable populations, deepening health inequities. Furthermore, climate change directly impairs health systems’ operations by damaging infrastructure, disrupting supply chains, and affecting the health workforce, thereby compromising service delivery.^6^ As climate change continues to create new health challenges and strain existing resources, health systems must adapt to protect and promote population health while simultaneously working to reduce their own contributions to environmental degradation and greenhouse gas emissions.^7^

In this context, health systems must evolve to mitigate the impact of climate change on health. A climate-resilient health system can anticipate, respond to, cope with, recover from, and adapt to climate-related shocks and stress, ultimately improving population health despite climate instability.^4^ To build such resilience, it’s crucial to understand the core components of health systems. The World Health Organization (WHO) health systems framework comprises five core dimensions: leadership and governance, health workforce, health information systems, essential medical products and technologies, and service delivery. These interconnected components form the foundation of a complex structure aimed at improving health outcomes and delivering healthcare services to populations.^4,5^ As climate change intensifies, it becomes increasingly crucial for these systems to adapt, not only to protect and promote population health but also to reduce their own environmental footprint. This dual challenge requires a holistic approach that integrates climate resilience and sustainability across all aspects of health system operations.

While the World Health Organization’s Operational Framework for Building Climate Resilient Health Systems^4^ provides a comprehensive foundation for understanding the core components needed for climate resilience in health systems, there is a critical need to examine how these building blocks have been addressed in real-world contexts. Despite the growing body of literature on climate change and health,^4,8^ less attention has been paid to synthesizing how academic research, especially case studies, has reported on the practical implementation and challenges of building climate-resilient health systems.

Furthermore, while the importance of equity in climate change adaptation has been recognized,^9^ there is a need to understand how equity considerations have been integrated into the implementation of climate-resilient health systems. This review seeks to highlight how case studies and other academic literature have addressed equity issues within the framework of climate-resilient health systems.

By conducting a comprehensive analysis of how research on climate change has been developed alongside health systems, with a particular focus on the WHO’s building blocks for climate-resilient health systems, this study aims to provide a critical overview of the current state of knowledge and practice. Our research question, “How has research concerning climate change and health is connected with global frameworks for climate resilient health systems?,” seeks to uncover not only what is known, but also to identify crucial knowledge gaps and areas for future investigation in the practical implementation of climate-resilient health systems.^10^ This synthesis may contribute to a better understanding of how the theoretical framework for climate-resilient health systems has been translated into practice, identifying successful strategies, common challenges, and areas needing further attention.^5,11^ Ultimately, this review aims to inform more climate-resilient health systems capable of addressing the complex and evolving health needs of diverse populations in the face of climate change.

## Background

### Climate change impacts on population health

The direct health impacts of climate change include increased morbidity and mortality due to extreme weather events, such as heatwaves, floods, and hurricanes, which can lead to injuries, deaths, and the exacerbation of chronic health conditions.^8^ The World Health Organization estimates that climate change could cause approximately 400 000 deaths annually between 2030 and 2050 due to its effects on health systems and social determinants of health.^12^ For example, heat-related illnesses are expected to rise as global temperatures increase, particularly affecting vulnerable populations such as the elderly and those with pre-existing health conditions.^13^ Indirectly, climate change influences health through environmental changes that affect air quality, water supply, and food security, leading to respiratory diseases, waterborne illnesses, and malnutrition.^8^ Moreover, mental health consequences are increasingly recognized, as climate change can lead to anxiety, depression, and post-traumatic stress disorder due to displacement and loss of livelihoods.^14^

Climate change exacerbates existing health inequities, disproportionately affecting vulnerable populations.^8^ These groups face higher risks from extreme weather events, infectious diseases, and food and water insecurity. Low-income countries are particularly vulnerable due to limited resources and weak infrastructure. In sub-Saharan Africa, where over 60% of the population relies on rain-fed agriculture, climate-related droughts and flooding can lead to crop failures, food shortages, and malnutrition, increasing susceptibility to diseases like malaria.^15^ Indigenous communities in remote areas also face significant risks; for instance, in the Arctic, melting sea ice has led to the loss of traditional food sources, causing food insecurity and increased risk of chronic diseases.^16^ The impacts are also present in global north countries, where older individuals and those with chronic health conditions are at higher risk. More frequent heat waves exacerbate respiratory and cardiovascular conditions in the elderly, resulting in increased hospitalizations and deaths.^17^

### Health systems as mediators of climate change effects

Climate change poses significant challenges to health systems worldwide, testing their resilience and exacerbating health inequities. The Intergovernmental Panel on Climate Change (IPCC)^18^ reports that extreme weather events such as floods, hurricanes, and wildfires can severely damage health facilities, causing service interruptions and loss of critical medical equipment. This issue is particularly acute in low-income countries with already inadequate health infrastructure.^19^ Moreover, climate change intensifies existing workforce shortages by increasing climate-related illnesses and forcing health workers to relocate,^20^ potentially compromising healthcare delivery, especially in regions with weak healthcare systems.^21^

The health workforce is crucial in addressing the health impacts of climate change, yet it faces significant challenges. The increasing demand for health services due to heat-related illnesses and vector-borne diseases necessitates a workforce that is not only adequately trained but also sufficiently staffed.^22^ This leads to a call for the integration of climate change competencies into medical education, as a critical aspect for equipping future healthcare professionals with the necessary skills to address climate-related health challenges.^23^ The mental health implications of climate change are particularly concerning, with issues such as eco-anxiety, post-traumatic stress disorder from extreme weather events, and depression related to climate displacement becoming increasingly prevalent.^24^ Healthcare professionals need to be trained to recognize and manage these psychological impacts, as well as understand the complex interplay between climate change, mental health, and social determinants of health.^25^

To foster climate-resilient health systems, it is imperative to implement effective adaptation strategies. This includes integrating climate change considerations into health service planning and policy-making and incorporating adaptive management principles into public health practice.^26^ Local governments are crucial in this adaptation process, as they are often the first responders to climate-related health challenges. Local government environmental health officers possess the expertise necessary to develop and implement adaptation plans that protect public health in the face of climate change.^27^ By fostering collaboration between health systems, local governments, and communities, it is possible to create a more integrated and effective response to the health impacts of climate change.

## Methods

**Figure 1.**
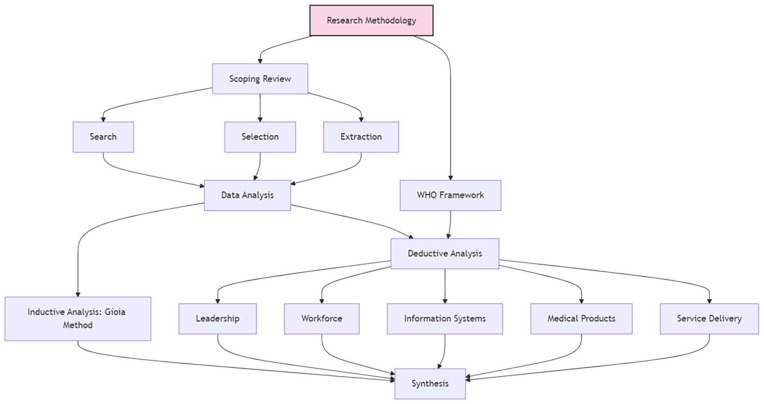
Methods walkthrough.

This study employed a scoping review method to investigate the topic at hand. The review process followed four main steps: (1) Search strategy and information sources, (2) Study selection, (3) Data extraction and synthesis, and (4) quality assessment and bias consideration. To provide a clear overview of our methodological approach, we have created a Methods Walkthrough (Figure 1) that visually represents the steps taken in this scoping review.

### Search strategy and information sources

The analysis sources comprise the following academic databases: PubMed, Scopus and Web of Science. These databases were chosen due to their comprehensive coverage of science literature. Two search strings were developed by combining climate change-related keywords and health systems-related keywords. The Boolean operator “AND” was used to connect both strings, while the Boolean operator “OR” was used within each string. The search was conducted in April 2023. The full search strings and parameters are presented in Table 1.

**Table 1.** Search strings.

(1) *Climate change-related keywords*	
Global warming OR Climate crisis OR Climate disruption OR Climate variability OR Climate shift OR Climate instability OR Climate perturbation OR Climate abnormality OR Climate anomaly OR Climate fluctuation OR Anthropogenic climate change OR Human-induced climate change OR Climate emergency OR Climate catastrophe OR Climate degradation OR Climate deterioration OR Climate alteration OR Climate modification OR Climate manipulation OR Climate transformation OR Climate variability and change OR Climate shift and adaptation OR Climate anomalies and trends OR Climate unpredictability OR Climate hazards and risks OR Climate sensitivity and feedbacks OR Climate dynamics and modeling OR Climate extremes and events OR Climate-induced disasters OR Climate resilience and vulnerability OR Climate mitigation and adaptation strategies OR Climate policy and governance OR Climate justice and ethics OR Climate communication and education OR Climate action and activism OR Climate finance and investment OR Climate science and research OR Climate impacts and consequences OR Climate feedback mechanisms OR Climate system drivers	
(2) *Health systems related keywords*	
“Health system* OR Healthcare system* OR Health-care system* OR Healthcare delivery OR Health-care delivery OR Healthcare service* OR Health-care service* OR Health service* OR Healthcare infrastructure OR Health-care infrastructure OR Medical systems OR Public health system* OR Public Health OR Electronic health records OR Healthcare workforce OR Health policy OR Healthcare quality OR Healthcare financing”	
#	*Exclusion criterion*
EC1	Non-English written papers
EC2	Duplicated studies (only one copy of each study was included)
EC3	Excluded document types that are not peer-reviewed articles
#	*Inclusion criterion*
IC1	Approaches of climate change alongside health systems

### Study selection

Inclusion criteria focused on peer-reviewed, English-language studies addressing both climate change and health systems. The selection process involved title/abstract screening followed by full-text review, conducted independently by the first author and two reviewers. Disagreements were resolved through discussion or third-party consultation. The research flow, including the number of articles selected at each step, can be seen in Figure 1. The results were narrowed from an initial sample of 3511 to 179 articles for full analysis.

### Data extraction and synthesis

Data extraction was conducted using a standardized form capturing key study characteristics, methods, findings, and health system domains addressed. The collected data were then synthesized using a two-phase approach:

(1) *Inductive Analysis*: We first employed a thematic analysis approach inspired by the Gioia methodology.^28^ This method involves a systematic, multi-step process:
A. *First-order analysis*: Identifying key concepts and themes directly from the extracted data
B. *Second-order analysis*: Grouping similar first-order concepts into broader categories
C. *Aggregate dimensions*: Consolidating second-order themes into overarching dimensions

This approach allowed for the identification of common themes and patterns across the included studies, while maintaining a close connection to the original data. This inductive approach allowed for the identification of common themes and patterns across the included studies, while maintaining a close connection to the original data, without being constrained by predetermined categories.^29^

(2) *Deductive framework application*: Following the inductive analysis, we used the World Health Organization (WHO) health systems framework^4^ to organize and further synthesize our findings. This framework provides a comprehensive and globally recognized structure for understanding and analyzing health systems, making it particularly suitable for our scoping review.
  1. Leadership and governance.
  2. Health workforce.
  3. Health information systems.
  4. Essential medical products and technologies.
  5. Service delivery.

By applying this framework to our inductively derived themes, we were able to systematically categorize and analyze the literature, ensuring a comprehensive examination of how climate change impacts and is addressed by different aspects of health systems. This two-phase approach allowed us to both capture emerging themes from the literature and situate them within a well-established health systems framework.

### Quality assessment and bias consideration

While formal quality assessment is not typical in scoping reviews, we considered aspects such as study design clarity, methodological robustness, and relevance of findings. We acknowledged and attempted to mitigate potential biases, including selection bias, language bias, and publication bias. The PRISMA-ScR guidelines were followed to ensure transparent reporting (Appendix 1).

## Results

### Overview

**Figure 2.**
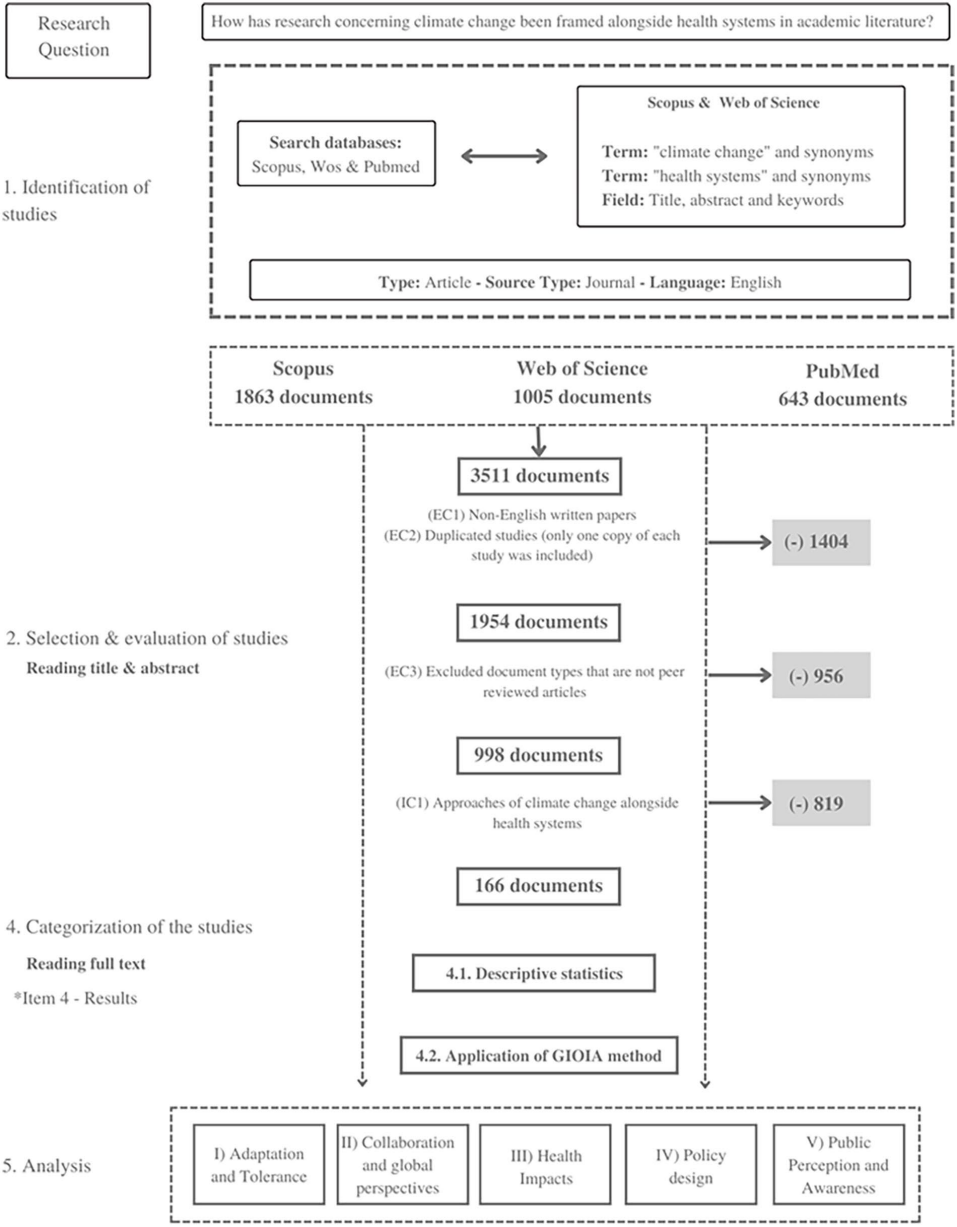
Research flow.

The initial search across three major databases (Scopus, Web of Science, and PubMed) yielded 3511 documents (1863 from Scopus, 1005 from Web of Science, and 643 from PubMed). After removing non-English papers and duplicates (EC1 and EC2), 1954 documents remained. Following the exclusion of non-peer-reviewed articles (EC3), 998 documents were retained. Finally, after applying our inclusion criterion of approaches to climate change alongside health systems (IC1), 179 documents were selected for full analysis. These papers were analyzed, leading to the identification of five main thematic areas: adaptation and tolerance, collaboration and global perspectives, health impacts, policy design, and public perception and awareness. The subsequent analysis was structured according to four key health system components: resource generation, governance, service delivery, and finance (Figure 2).

**Figure 3.**
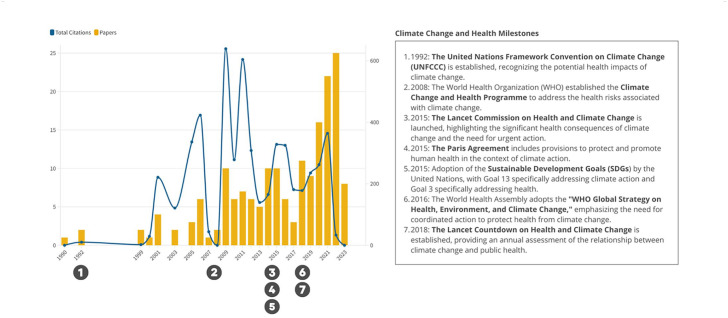
Scientific output over time.

Over time, the scientific output on climate change and health systems has evolved, with notable milestones marking the trajectory of research (Figure 3). The earliest publication, Longstreth^30^ highlighted the need to consider the health consequences of global warming alongside environmental effects. Building on this foundation, recent studies such as Somani^31^ and Arnot et al^32^ have explored the disproportionate health impacts on vulnerable populations in countries like Pakistan and the perspectives of young people on the political determinants of the climate crisis. The year 2009 marked the peak of citations, as demonstrated by Malka et al,^33^ examining the association between knowledge and concern about global warming. As the field progresses, 2022 emerges as a significant year with a peak in publications, exemplified by Nigussie et al^34^ investigating the spatiotemporal distribution of malaria incidence. These milestones reflect the growing recognition of climate change’s implications for human health and highlight the need for continued research, interdisciplinary collaboration, and targeted interventions to mitigate the health risks associated with climate change.

Major climate change policy milestones have significantly shaped health systems development. Patterson’s^35^ research examines how the United Nations Framework Convention on Climate Change (UNFCCC) influences state obligations and civic participation in health system responses. Maibach et al^14^ emphasize the Paris Agreement’s importance in advancing equitable health policies and system resilience. Máté et al^36^ connect these developments to sustainable development goals (SDGs), showing how climate policies affect health systems through their impact on economics, emissions, and life expectancy. Together, these studies demonstrate how international climate agreements have shaped health system evolution, particularly in areas of public health policy and social equity.

**Figure 4.**
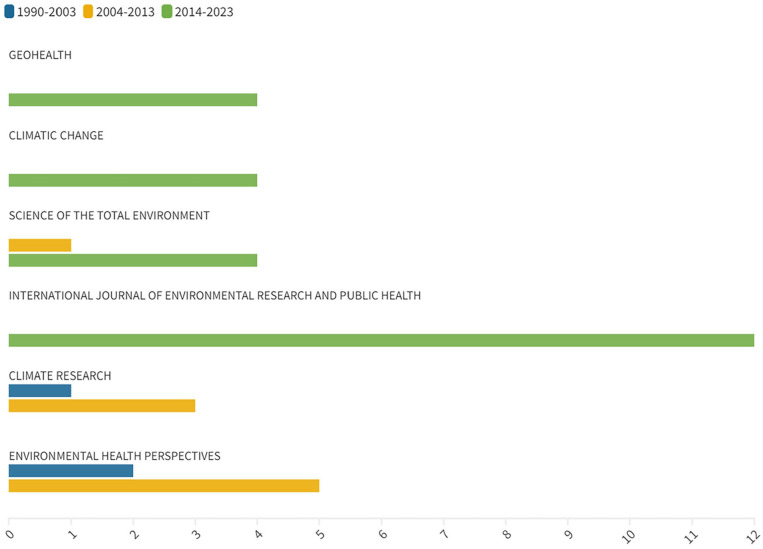
Main publication venues.

The field of climate change and health systems research is well represented by a diverse range of publications, spread across 132 different journals (Figure 4). One prominent source is the *International Journal of Environmental Research and Public Health*, which has contributed significantly with 12 publications. Earlier studies, before the Paris Agreement and published in journals such as *Environmental Health Perspectives and Climate Research*, have also made noteworthy contributions. For instance, Greenough et al^37^ examine the potential impacts of climate variability and change on the health effects of extreme weather events, while Méndez-Lázaro et al^38^ explore the relationship between climate variability and dengue incidence in San Juan, Puerto Rico. These studies show the importance of comprehending physiological constraints, enhancing climate models, and implementing effective public health interventions in the face of climate change.

### Country authorship and contributions

**Figure 5.**
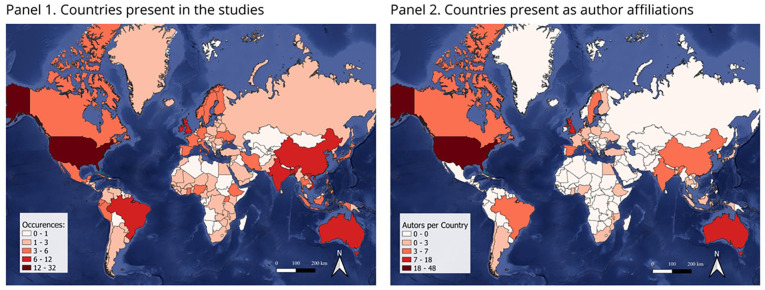
Countries described in the studies and author affiliations.

Our analysis of authorship and study locations reveals a complex landscape of global research on climate change and health systems (Figure 5). While there is a broad geographical distribution of contributions, it’s important to note that this distribution is not uniform and may not necessarily indicate true global collaboration or balanced involvement

The United States and the United Kingdom dominate the research output, with 48 and 11 instances in author affiliations respectively, potentially reflecting their strong research infrastructure and funding capabilities rather than inherent expertise in climate-health issues. The presence of “Global” in 38 instances of author affiliations suggests multi-country involvement, but this could be due to increased funding for global research projects rather than organic collaboration. While it’s encouraging to see contributions from developing nations like Bangladesh, Ethiopia, and Malawi, as well as emerging economies like Brazil, India, and Indonesia, their representation is still limited compared to Western countries. This imbalance raises questions about the global applicability of research findings and the potential overlooking of region-specific challenges. Future research efforts should aim to address these disparities, fostering more equitable participation and ensuring that the unique perspectives and needs of underrepresented regions are adequately captured in the global discourse on climate change and health systems.

### Main topics

The results of this study are organized into six main interconnected themes that explore the various effects of climate change on health systems, as presented in Table 2. These themes emerged from our analysis: adaptation and tolerance, collaboration and global perspectives, health impacts, policy design, public perception and awareness, and socio-ecological nexus.

(1) Adaptation and tolerance remains crucial for protecting public health, particularly regarding heat and environmental stresses. Recent studies demonstrate diverse health impacts requiring targeted interventions. In the Gaza Strip, research has shown increased diarrheal disease risk, particularly affecting young children (0-3 years).^39^ Climate impacts extend to cognitive development, with Tang and Di^40^ revealing how prenatal exposure to climate anomalies can affect adult cognitive function and economic outcomes. Neurological conditions show varying vulnerability to climate effects: Bongioanni et al^41^ found that Parkinson’s disease patients are particularly susceptible to heat stress, while those with Alzheimer’s and other dementias showed less sensitivity.
(2) Our analysis revealed strong interconnections between Collaboration and Global Perspectives and Health Impacts. The Nature Step to Health—Lahti Regional Health and Environment Programme 2022 to 2032 exemplifies organizations working together to promote planetary health.^42^ This collaborative approach is essential as climate change creates significant health risks, including extreme weather events, temperature-related illnesses, and vector-borne diseases.^43^ Studies estimate that around 15% of emergency room visits for heat-related illnesses in North Carolina are due to climate change,^44^ while the connection between climate change and high incarceration rates worsens health inequities.^45^
(3) Policy Design emerges as a critical factor in addressing these challenges. Wheeler et al^46^ emphasize the need for governments to plan responses to the climate crisis, especially for maternal and newborn health. This connects directly to Public Perception and Awareness, where studies by Arnot et al^32^ and Clery et al^47^ highlight the importance of medical activism and youth engagement. Kearney and Bell^48^ found that the poorest counties in the southeastern United States have a lower belief in global warming, emphasizing the need for targeted awareness campaigns.
(4) The final theme, the Socio-ecological Nexus, ties these elements together by emphasizing the connections between climate change, health systems, and various elements of the environment. Rising global temperatures increase risks of vector-borne diseases like dengue fever,^49^ while human-caused climate change has been identified as a key factor in the increase of wildfires.^50^ Higher levels of CO_2_ in the atmosphere directly impact plant biology and allergen exposure,^51^ demonstrating the complex interconnections between environmental and human health factors. These themes collectively illustrate the multifaceted nature of climate change impacts on health systems, highlighting the need for integrated approaches that consider both environmental and social factors in developing effective responses to these challenges.

**Table 2.** Categorization of topics.

First order topics (examples of paper topics)	Quotes from papers	Second order themes	Detail	Aggregate dimensions
Human heat tolerance and adaptation Maintaining and improving the public health infrastructure Building codes, warning systems, and disaster policies	*Human heat tolerance and adaptation* “The effects of temperature exposure on disease were mostly immediate, which suggests an acute mechanism of association, such as increased consumption of contaminated water or increased food spoilage during hot weather; this could inform potential behavioral changes whenever high temperatures are forecast.”^39^ “Our study also gives people hope: provinces developed, on the contrary, can use advantages in these aspects to compensate the impact of unfavorable climate, and similarly, policymakers can leverage economic development to reduce or even eliminate the impact of abnormal climate.”^40^ *Maintaining and improving the public health infrastructure* “Therefore, adaptation policies that target such groups may yield particularly high benefits.”^52^ “This paper describes a framework for conceptualizing adaptation within a social systems behavior change perspective, and a mechanism by which governments can better plan and gauge possible effects of their policy decisions, particularly regarding the behavior strategies employed.”^53^ *Building codes, warning systems, and disaster policies* “At the local level, natural disasters and climate change were rarely differentiated, and when it comes to risk management, it makes little practical sense to do so. However, participants noted that the integration, or sometimes conflation, of DRR and CCA in such a disaster-prone country has also meant that long-term climate change was often neglected”^54^ “It is necessary to develop an early warning framework and take the required action at both policy and healthcare service delivery levels to ultimately reduce hospital LOS and lessen the economic and health burden in a climate-vulnerable country such as Bangladesh.”^55^	Adaptation and tolerance	Human heat tolerance and adaptation strategies to mitigate the health effects of climate change, including measures to maintain and improve public health infrastructure	Adaptation and tolerance
Collaboration and global perspectives Government and non-governmental organizations’ role in disaster response	*Collaboration and global perspectives* “Joining an organization that focuses specifically on climate and health is perhaps the easiest way for health professionals to align themselves with others seeking to advance climate and health solutions. Examples of such organizations include alliance of nurses for a healthy environment; global climate and health alliance; medical society consortium for climate and health; and physicians for social responsibility.”^14^ “Altogether, these findings emphasize the need for urgent coordinated national and international interventions to limit antimicrobial use and the global spread of AMR.”^56^ *Government and non-governmental organizations’ role in disaster response* “Bed net distribution was scaled-up in the study area and other malaria-endemic areas in Kenya during the period between 2006 and 2014. It was observed that an increase in bed net use had an important protective effect, especially during 2013-2015. Similar findings were reported elsewhere”^57^ “Although 634,000 people have been moved into temporary shelters created by the government and non-government organizations, they are still facing serious problems. In the relief camps, people are fighting with infectious diseases, including diarrhea, skin infections, and eye diseases.”^31^	Collaboration and global perspectives	Collaboration and global efforts to address climate change and its health impacts, including the role of government and non-governmental organizations in disaster response and the need for global perspectives.	Collaboration and global perspectives
Geographic and temporal variability in health impact	*Geographic and temporal variability in health impact* “The more frequent occurrence of these two compound events in a warming climate revealed from this study may affect both natural and human systems. . . For instance, the higher frequency of CDWEs in large regions may cause reduced crop productions and risk global food security. . . Further, the increased compound wet-warm events may negatively affect public health”^58^ “A changing climate with thawing permafrost, in turn, threatens infrastructure, food and water resources, and human health, especially for the indigenous peoples in the Arctic region, who live in permafrost areas and depend on local resources.”^59^	Geographic and temporal variability	Geographic and temporal variability in health impacts, including regional variations in the health effects of climate change.	
Health impacts of extreme weather events	*Health impacts of extreme weather events* ‘“Impacts of climate change, such as extreme weather, threaten to further exacerbate the eroding maternal and newborn health gains, compounding existing gaps and amplifying global and national failures. Marginalized communities, already struggling with the highest rates of maternal and newborn morbidity and mortality, are most vulnerable to the effects of changing climate.”^46^ “Our findings suggest about 3 out of 20 HRI emergency department visits in North Carolina were attributable to anthropogenic climate change. [. . .] Heat-related morbidity is 3.69 times higher than the heat-related mortality rate.”^44^	Health impacts	The health consequences of climate change, including the impacts of extreme weather events, temperature-related morbidity and mortality, vector-borne diseases, waterborne diseases, and the burden of disease transmission.	Health impacts
Temperature-related morbidity and mortality in the U.S. Mortality burdens associated with climate change	*Temperature-related morbidity and mortality in the U.S.* “Our results suggest that heat-related morbidity is 3.69 times higher than the heat-related mortality rate reported by Vicedo-Cabrera et al. (2021). The higher number of HRI could be due to the difference in the total number of cities included in our study. . . It is also natural that morbidity should be higher than estimates of mortality, as not every heat-related illness results in a death.”^46^ “Studies have shown mental health impacts following floods and evacuations. The increase in temperature will result in a higher risk of heatstroke and heat-related distress in connection with physical activity, which may have major occupational health consequences.”^43^ *Mortality burdens associated with climate change* “Several researchers have recently argued that such an ability to robustly attribute specific damages to anthropogenic drivers of increased extreme heat can inform societal responsibilities for the costs of both ‘loss and damage’ and adaptation in developed as well as developing countries.”^60^ “In this large nationwide study among a long-lived Chinese elderly population, we showed for the first time that extreme ENSO exposure was capable of heightening mortality risks and medical burden in the last year of life.”^61^	Mortality burdens	The mortality burdens associated with climate change, such as heat-related mortality and the influence of climate change on infectious disease outbreaks.	
Policy and research	“Even during the ‘good times’ of the past few decades, public health policy and research on global health problems have been poorly coordinated, underfunded and have done little to benefit 90% of the world’s poorest population.”^62^ “More important, given the current and projected health risks of climate change in the United States, Congress needs to allocate funds to federal agencies whose mission mandates include human health; these agencies should maintain and enhance programs (and appropriate funding) to specifically address climate change risks in a timely and efficient manner.”^8^	Policy and research	Funding for climate change and health research, as well as probabilistic projections and decision support for climate regime shifts.	Policy design
Public perception and awareness: Knowledge, concern, and public perception of global warming Public awareness of health effects of global warming	*Knowledge, concern, and public perception of global warming* “This study also demonstrated that young people perceived that climate policies were influenced by powerful elites and corporate actors. Public health advocacy may provide a pathway in which young people feel empowered to act, and a mechanism for channeling frustration, ideas, and energy into effective strategies to influence decision-makers.”^32^ “There is irrefutable scientific evidence of profoundly detrimental physical and mental health outcomes due to the [Climate and Ecological Emergency (CEE)]. As the doctor, educator and therapist John Launer reflects: ‘As health and social inequalities widen, and we learn more about the power held over human lives by an ever-diminishing number of individuals and corporations, I predict more doctors around the world will be drawn inescapably into political campaigning.’”^47^ *Public awareness of health effects of global warming* “Participants who had or wanted to have children tried to find a balance between ethically competing goods, through living a more climate-friendly family life or by having fewer children. . . Participants who did not have or want children balanced competing goods, too. Some indicated that being childfree could allow them to do other things that would bring them joy but increase their carbon footprint, such as doing more airplane travel.”^63^ “Studies have revealed that nursing students have high anxiety levels about the environment and they will be worried if global diseases emerge. One reason for this may be the fact that the data was collected during the COVID-19 pandemic. Senior nursing students listed skin diseases, cardiovascular diseases, water-borne diseases, and, to a large extent, respiratory system diseases when they were asked about the effects of global warming on health.”^64^	Public perception and awareness	Knowledge, concern, and public skepticism about global warming, as well as public awareness of the health effects of global warming.	Public perception and awareness
Audience segmentation for climate change communication	“Participation enables the advancement of all human rights. It plays a crucial role in the promotion of democracy, the rule of law, social inclusion and economic development. It is essential for reducing inequalities and social conflict. It is also important for empowering individuals and groups, and is one of the core elements of human rights-based approaches aimed at eliminating marginalization and discrimination.”^35^	Climate change communication	Audience segmentation for climate change communication to effectively convey the health risks associated with climate change.	
*Effects of climate change on environmental contaminants and human health* Anthropogenic climate change and wildfires	*Effects of climate change on environmental contaminants and human health* “Quantitative comparisons between health care workers and plant biologists as to what constitutes pollen season and subsequent cooperation to establish a common set of plant biological indices in regard to climate would be very useful in this regard.”^51^ “One of the first questions we asked was: to what extent will climate change affect future air pollution, holding everything else constant? And given those changes, what would be the health impacts? I had a special interest in ground-level ozone, which gets worse when temperatures increase.”^65^ *Anthropogenic climate change and wildfires* “Climate change will increase summertime surface ozone levels in polluted regions, with the largest effects in urban areas during pollution episodes; in addition, climate change–enhanced wildfires could become an increasingly important particulate matter (PM) source.”^51^ “Ozone formation is greater at higher temperatures, and ozone is the main component of summer smog episodes.”^51^	Environmental contaminants	The effects of climate change on environmental contaminants and their impact on human health.	Socio-ecological nexus
Effects of elevated CO_2_ on plant biology and human health	“Recent and projected increases in CO_2_ concentrations could increase the carbon to nitrogen ratio in leaves of timothy grass (*Phleum pratense*), with subsequent increases in the onset of sporulation of *Alternaria alternata*.”^51^	Plant biology and CO_2_ effects	The effects of elevated CO_2_ on plant biology and its implications for human health	
Anthropogenic climate change substantially amplified the risk factors by extreme wildfire season	“Our analysis demonstrates that anthropogenic climate change has significantly increased the likelihood of extreme wildfire events. Specifically, the observed 2017 wildfire season in British Columbia, which saw unprecedented burned areas and prolonged emergency states, was substantially influenced by human-induced warming. The increased probability of extreme temperatures and elevated wildfire risk, as well as the substantial area burned, can be directly attributed to anthropogenic climate change.”^50^	Wildfires and anthropogenic climate change	The relationship between anthropogenic climate change and wildfires and their health implications.	
Climate change and insecticide exposure in mosquito-borne diseases	“Research on the potential impact of global warming on malaria is of high interest for public health. Recently, insecticides used for malaria vector control have been tested under varying temperatures (18°C, 25°C, and 30°C) against adults of *Anopheles arabiensis* and *Anopheles funestus*. Higher temperatures boosted the resistance of susceptible *A. arabiensis* to deltamethrin, while exposing susceptible *A. funestus* and resistant *A. arabiensis* females to deltamethrin under temperatures above and below 25°C led to a mortality increase.”^66^	Insecticide exposure and mosquito-borne diseases	The impact of climate change on insecticide exposure and the spread of mosquito-borne diseases.	

## Discussion

This section examines the current literature on climate change and health systems through the lens of the WHO framework for climate-resilient and low-carbon health systems. As outlined in our methods, the WHO health systems framework comprises five core components: leadership and governance, health workforce, health information systems, essential medical products and technologies, and service delivery. These components form the foundation of health systems at various scales, from local clinics to national healthcare networks. Our analysis reveals that climate change affects these components in distinct ways, as evidenced by the main topics identified in our results section.

The impact of climate change on these components can vary based on the type and scale of the health system, as well as geographical and socioeconomic factors, as demonstrated by studies in diverse settings such as the Gaza Strip, Ethiopia, and the southeastern United States. In the following subsections, we analyze how each of these components is affected by climate change, synthesize the current state of research, and identify gaps in our understanding.

### Climate-transformative leadership and governance

Climate-transformative leadership and governance is key for addressing the complex interplay between climate change and health systems. As highlighted in our results on “Collaboration and global perspectives” and “Policy design,” the framework stresses integrating climate change issues into health system planning and decision-making processes,^46,67^ emphasizing cross-sector collaboration and stakeholder participation.^32,42^ However, major challenges exist in implementation, and effective collaboration is often hindered by separate governmental structures and different priorities.

### Climate-smart health workforce

The climate-smart health workforce component recognizes the importance of human resources in building climate resilience and environmental sustainability. As our results on “Adaptation and Tolerance” and “Health Impacts” show, the health sector faces significant challenges in developing specialized skills for managing climate-related health risks.^44^ Putting into practice workforce development goals is hindered by limited resources for training, competing priorities in health education programs, and the need for quick upskilling of the existing workforce. The study by Thiel et al,^68^ on obstetricians and gynecologists’ willingness to participate in sustainability efforts demonstrates the potential for physician involvement, but also highlights the need for more comprehensive training and support.

### Health information systems

The health information systems component is crucial for evidence-based decision-making in climate-resilient health systems. Our results on “Collaboration and global perspectives” and “Health Impacts” underscore the importance of integrating climate data with health surveillance systems.^39^ However, putting into practice integrated climate and health information systems faces significant challenges, including data quality issues, problems connecting different information systems, and the need for advanced analytical capabilities that may be missing in many health systems. The study in the Amhara region of Ethiopia^34^ demonstrates the complexity of integrating climate data with health information, particularly in resource-limited settings.

### Essential medical products and technologies

This component addresses the important aspect of adapting health system infrastructure and technologies to climate change. As highlighted in our “Health Impacts” and “Policy design” sections, the move to low-carbon technologies and sustainable supply chains requires significant upfront investment and may compete with other pressing health system needs.^43^ While the framework correctly identifies potential for GHG emissions reductions in health care supply chains, it may underestimate the financial and practical barriers to implementation. The study by Nakajima et al^69^ on short-lived climate pollutants (SLCPs) illustrates the complexity of balancing immediate health needs with long-term climate mitigation strategies.

### Service delivery

**Figure 6.**
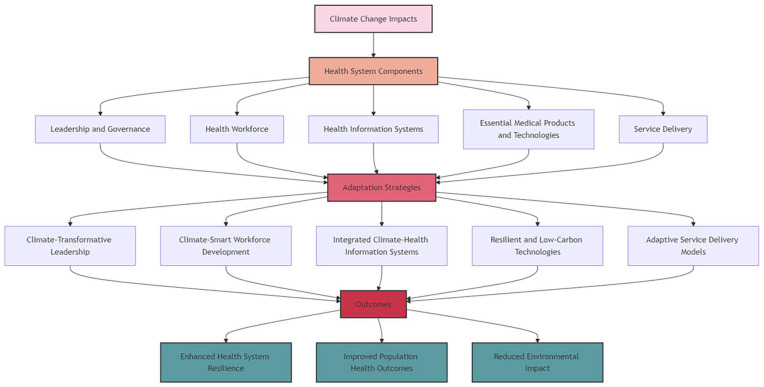
Integrated summary framework.

The service delivery component is crucial for turning climate resilience strategies into real health outcomes. Our results on “Adaptation and Tolerance” and “Health Impacts” demonstrate the urgent need for adapting health services to changing climate conditions.^40,44^ However, putting into practice climate-informed health programs faces significant challenges, including limited resources, competing health priorities, and the need for complex, multi-sector interventions. Moreover, the increasing frequency and intensity of climate-related health emergencies may overwhelm health system capacity, particularly in vulnerable regions. The research in Palestine provides an example of how climate change can exacerbate existing service delivery challenges, particularly in fragile settings.^39^ To synthesize our findings and illustrate the interconnections between various aspects of climate change impacts on health systems, we have developed an Integrated Summary Framework (Figure 6). This framework visually represents the key themes identified in our review and their relationships to the WHO’s climate-resilient health systems components

## Conclusion

This study helps us better understand the complex relationship between climate change and health systems, showing the need for proactive steps to reduce negative health effects. Our findings stress the importance of adaptation strategies and improving infrastructure to address health risks from climate change. Specifically, we highlight the importance of measures to deal with heat and the need to maintain and improve public health infrastructure. These adaptation strategies are crucial to reduce the impact of climate change on health, especially for vulnerable groups.

Working together and having global perspectives are key in addressing the challenges of climate change and health systems. Programs like the Nature Step to Health show the importance of organizations working together to promote planetary health and include climate change considerations in health programs. Our study also points out the major health impacts of climate change, including extreme weather events, illnesses related to temperature, and increased disease burdens. These findings underscore the need for evidence-based actions, efforts to reduce carbon emissions, and a focus on reducing greenhouse gasses. Good policy design, public awareness, and including climate change considerations in health systems are crucial for maintaining and improving public health infrastructure. By recognizing the connections between climate change, health systems, and the environment, we can develop comprehensive approaches to reduce negative health effects and build resilience to environmental changes.

### Policy implications

Public health agencies play a crucial role in climate-transformative leadership, and policymakers must prioritize strengthening these institutions to effectively address the challenges posed by climate change. This includes increasing funding and resources for climate-related health monitoring and response, developing training programs for public health professionals, and establishing clear mandates for integrating climate change considerations into planning and operations.^14^ Public health agencies are uniquely positioned to assess health risks, develop adaptation strategies, and implement protective policies for vulnerable populations. They can also serve as effective messengers, raising awareness about the health implications of climate change and quantifying the health co-benefits of climate actions to inform policy decisions.^70,71^ To leverage this potential, policymakers should create robust communication channels between public health agencies and decision-makers, ensuring consistent integration of health considerations into climate policy.

Overcoming implementation challenges is critical for effective climate-health adaptation. Policymakers should address the barriers faced by public health agencies by establishing clear governance structures and responsibilities across different government levels, allocating dedicated funding for climate-health adaptation initiatives, and promoting knowledge sharing among agencies.^72^ To support proactive risk management, policymakers should mandate the inclusion of climate change scenarios in public health assessments and provide comprehensive guidelines for climate vulnerability assessments and adaptation planning. Additionally, recognizing the economic implications of climate change on health, policymakers should incorporate health economic analyses into climate policy decision-making and invest in research to quantify these impacts.^73^ By implementing these policies, public health agencies can more effectively prepare for and mitigate the health impacts of climate change, contributing to more resilient and adaptive health systems.

#### Reframing climate change as a health issue

One of the most significant policy implications is the need to reframe climate change as a public health issue. This perspective can enhance public awareness and support for climate policies by emphasizing the co-benefits of climate action, such as improved air quality and healthier communities.^74^ Research indicates that when climate change is framed within the context of health, it resonates more effectively with the public, leading to increased support for mitigation strategies.^75^ Policymakers should prioritize communication strategies that highlight these health benefits, thereby fostering a more robust public dialogue around climate action.^76^

#### Strengthening health systems for climate adaptation

Investments in health systems are crucial for building resilience against climate change impacts. Policymakers must allocate adequate resources to strengthen health infrastructure, ensuring that it can withstand climate-related stresses and shocks.^7^ This includes not only financial investments but also the development of training programs for healthcare professionals to equip them with the necessary skills to address climate-related health risks.^14^ Furthermore, integrating traditional and modern health practices can enhance the adaptability of health systems in resource-poor settings.^21^

#### Enhancing collaboration across sectors

Effective climate action requires collaboration across various sectors, including health, environment, and urban planning. Policymakers should foster partnerships among stakeholders to create comprehensive strategies that address the multifaceted challenges posed by climate change.^9^ This collaborative approach can facilitate the sharing of resources and knowledge, ultimately leading to more effective implementation of climate-resilient health policies.^77^ Additionally, local governments are well-positioned to incorporate co-benefits into decision-making processes, as they can address immediate community needs while aligning with broader climate goals.^78^

#### Addressing mental health impacts

The mental health implications of climate change are increasingly recognized, necessitating that policymakers incorporate mental health considerations into climate action plans. Government initiatives should focus on reducing the distress associated with climate inaction, particularly among vulnerable populations such as youth. By prioritizing mental health in the context of climate change, policymakers can enhance overall community resilience and well-being.^79^

### Study limitations

While our study provides valuable insights, it’s important to note some limitations. Firstly, we mainly used existing literature and secondary data sources, which may introduce biases or miss some information. Future research should try to collect primary data and conduct empirical studies to strengthen the evidence. Also, our study gave a broad overview of health systems and climate change, and more detailed research into specific regions or health system contexts is needed to capture the complexities of these interactions. Finally, we didn’t look at the social, cultural, and behavioral aspects that influence how health systems respond to climate change, which could be an area for future research.

## Supplemental Material

sj-docx-1-ehi-10.1177_11786302241298789 – Supplemental material for The Double Burden: Climate Change Challenges for Health Systems Supplemental material, sj-docx-1-ehi-10.1177_11786302241298789 for The Double Burden: Climate Change Challenges for Health Systems by Flavio Pinheiro Martins, Marco Antonio Catussi Paschoalotto, Jose Closs, Meike Bukowski and Mariana M Veras in Environmental Health Insights

## Appendix 1

Full search string sample. *SCOPUS string sample.* (TITLE-ABS-KEY (“climate variability and change” OR “climate shift and adaptation” OR “climate anomalies and trends” OR “climate unpredictability” OR “climate hazards and risks” OR “climate sensitivity and feedbacks” OR “climate dynamics and modeling” OR “climate extremes and events” OR “climate-induced disasters” OR “climate resilience and vulnerability” OR “climate mitigation and adaptation strategies” OR “climate policy and governance” OR “climate justice and ethics” OR “climate communication and education” OR “climate action and activism” OR “climate finance and investment” OR “climate science and research” OR “climate impacts and consequences” OR “climate feedback mechanisms” OR “climate system drivers” OR “global warming” OR “climate crisis” OR “climate disruption” OR “climate variability” OR “climate shift” OR “climate instability” OR “climate perturbation” OR “climate abnormality” OR “climate anomaly” OR “climate fluctuation” OR “anthropogenic climate change” OR “human-induced climate change” OR “climate emergency” OR “climate catastrophe” OR “climate degradation” OR “climate deterioration” OR “climate alteration” OR “climate modification” OR “climate manipulation” OR “climate transformation” OR “climate change”) AND TITLE-ABS-KEY (“health systems” OR “healthcare systems” OR “healthcare delivery” OR “healthcare services” OR “health services” OR “healthcare infrastructure” OR “medical systems” OR “public health systems”)) AND (LIMIT-TO (PUBSTAGE, “final”)) AND (LIMIT-TO (DOCTYPE, “ar”)) AND (LIMIT-TO (LANGUAGE, “English”)) AND (LIMIT-TO (SRCTYPE, “j”))
