# Supplementary material for: The Double Burden: Climate Change Challenges for Health Systems
Source: Environ Health Insights. 2024 Nov 20;18:11786302241298789. doi: 10.1177/11786302241298789 (PMC11580064; doi:10.1177/11786302241298789)
Supplement: sj-docx-1-ehi-10.1177_11786302241298789 – Supplemental material for The Double Burden: Climate Change Challenges for Health Systems [file sj-docx-1-ehi-10.1177_11786302241298789.docx]

**Supplementary Data I - PRISMA_2020_checklist - The Double Burden: Climate Change Challenges for Health Systems**

*From:*  Page MJ, McKenzie JE, Bossuyt PM, Boutron I, Hoffmann TC, Mulrow CD, et al. The PRISMA 2020 statement: an updated guideline for reporting systematic reviews. BMJ 2021;372:n71. doi: 10.1136/bmj.n71

| **Section/Topic** | **Item #** | **Checklist item** | **Location where item is reported** |
| --- | --- | --- | --- |
| **TITLE** |  |  |  |
| Title | 1 | Identify the report as a systematic review. | The paper is identified as a scoping review, which is a type of systematic review. Title could be: "The Double Burden: Climate Change Challenges for Health Systems - A Scoping Review" |
| **ABSTRACT** |  |  |  |
| Abstract | 2 | See the PRISMA 2020 for Abstracts checklist. | The abstract effectively covers all key elements of an academic abstract in a concise manner. It provides context on climate change's impact on health systems, clearly states the paper's objective to analyze literature and identify themes, outlines the scoping review methodology, presents key findings on health system resilience, and concludes with implications for future research and interventions. This structure gives readers a comprehensive overview of the study's purpose, methods, results, and significance. |
| **INTRODUCTION** |  |  |  |
| Rationale | 3 | Describe the rationale for the review in the context of existing knowledge. | The introduction provides a rationale, discussing the impacts of climate change on health systems and the need for a comprehensive review. |
| Objectives | 4 | Provide an explicit statement of the objective(s) or question(s) the review addresses. | The research question is stated: "How has research concerning climate change and health is connected with global frameworks for climate resilient health systems?" |
| **METHODS** |  |  |  |
| Eligibility criteria | 5 | Specify the inclusion and exclusion criteria for the review and how studies were grouped for the syntheses. | Inclusion and exclusion criteria are mentioned in section "**Methods"**, including peer-reviewed English-language studies addressing both climate change and health systems. |
| Information sources | 6 | Specify all databases, registers, websites, organisations, reference lists and other sources searched or consulted to identify studies. Specify the date when each source was last searched or consulted. | PubMed, Scopus, and Web of Science were used. The search was conducted in April 2023. |
| Search strategy | 7 | Present the full search strategies for all databases, registers and websites, including any filters and limits used. | Search strings are provided in Table 1. |
| Selection process | 8 | Specify the methods used to decide whether a study met the inclusion criteria of the review, including how many reviewers screened each record and each report retrieved, whether they worked independently, and if applicable, details of automation tools used in the process. | The selection process involved title/abstract screening followed by full-text review, conducted independently by the first author and two reviewers. |
| Data collection process | 9 | Specify the methods used to collect data from reports, including how many reviewers collected data from each report, whether they worked independently, any processes for obtaining or confirming data from study investigators, and if applicable, details of automation tools used in the process. | Data extraction was conducted using a standardized form, conduct by the first author and two independent reviewers |
| Data items | 10a, 10b | List and define all outcomes for which data were sought. List and define all other variables for which data were sought. | The review extracted key study characteristics, methods, findings, and health system domains addressed. Specific variables are not exhaustively listed. |
| Study risk of bias assessment | 11 | Specify the methods used to assess risk of bias in the included studies, including details of the tool(s) used, how many reviewers assessed each study and whether they worked independently, and if applicable, details of automation tools used in the process. | Formal quality assessment is not typical in scoping reviews, but aspects such as study design clarity and methodological robustness were considered. |
| Effect measures | 12 | Specify for each outcome the effect measure(s) used in the synthesis or presentation of results. | Not applicable for this scoping review. |
| Synthesis methods | 13a-13f | Describe the processes used to decide which studies were eligible for each synthesis, methods to prepare data, tabulate or visually display results, synthesize results, explore causes of heterogeneity, and conduct sensitivity analyses. | A two-phase approach was used: 1) Inductive analysis using the Gioia methodology, 2) Deductive framework application using the WHO health systems framework. |
| Reporting bias assessment | 14 | Describe any methods used to assess risk of bias due to missing results in a synthesis (arising from reporting biases). | The authors acknowledged potential biases, including selection bias, language bias, and publication bias. |
| Certainty assessment | 15 | Describe any methods used to assess certainty (or confidence) in the body of evidence for an outcome. | Not applicable for this scoping review. |
| **RESULTS** |  |  |  |
| Study selection | 16a | Describe the results of the search and selection process, from the number of records identified in the search to the number of studies included in the review, ideally using a flow diagram. | Figure 2 provides a research flow diagram, showing the narrowing from an initial sample of 3511 to 179 articles for full analysis. |
|  | 16b | Cite studies that might appear to meet the inclusion criteria, but which were excluded, and explain why they were excluded. | Not applicable for this scoping review. |
| Study characteristics | 17 | Cite each included study and present its characteristics. | The paper cites several studies throughout, but a comprehensive list of all 179 included studies is not provided in the summary. |
| Risk of bias in studies | 18 | Present assessments of risk of bias for each included study. | Not applicable for this scoping review. |
| Results of individual studies | 19 | For all outcomes, present, for each study: (a) summary statistics for each group (where appropriate) and (b) an effect estimate and its precision (e.g. confidence/credible interval), ideally using structured tables or plots. | Not applicable for this scoping review. |
| Results of syntheses | 20a-20d | Present results of all syntheses conducted, including characteristics and risk of bias among contributing studies, results of statistical syntheses, investigations of heterogeneity, and sensitivity analyses. | The results are presented thematically under six main topics: Adaptation and Tolerance, Collaboration and Global Perspectives, Health Impacts, Policy Design, Public Perception and Awareness, and Socio-ecological Nexus. |
| Reporting biases | 21 | Present assessments of risk of bias due to missing results (arising from reporting biases) for each synthesis assessed. | Not applicable for this scoping review. |
| Certainty of evidence | 22 | Present assessments of certainty (or confidence) in the body of evidence for each outcome assessed. | Not applicable for this scoping review. |
| **DISCUSSION** |  |  |  |
| Discussion | 23a | Provide a general interpretation of the results in the context of other evidence. | The discussion section interprets the findings in the context of the WHO framework for climate-resilient and low-carbon health systems. |
|  | 23b | Discuss any limitations of the evidence included in the review. | Limitations are discussed, including the geographical imbalance in research and the challenges in implementing climate-resilient health systems. |
|  | 23c | Discuss any limitations of the review processes used. | Limitations of the study are acknowledged, including potential biases from using mainly secondary data sources and the broad overview approach. |
|  | 23d | Discuss implications of the results for practice, policy, and future research. | Implications for policy and practice are discussed, including the need for climate-transformative leadership and increased funding for climate-health research. |
| OTHER INFORMATION |  |  |  |
| Registration and protocol | 24a | Provide registration information for the review, including register name and registration number, or state that the review was not registered. | Not applicable for this scoping review. |
|  | 24b | Indicate where the review protocol can be accessed, or state that a protocol was not prepared. | Protocol is available as "Supplmentary Data I - PRISMA Checklist" |
|  | 24c | Describe and explain any amendments to information provided at registration or in the protocol. | None |
| Support | 25 | Describe sources of financial or non-financial support for the review, and the role of the funders or sponsors in the review. | No funding was used for |
| Competing interests | 26 | Declare any competing interests of review authors. | Authors declare no competing interests |
| Availability of data, code and other materials | 27 | Report which of the following are publicly available and where they can be found: template data collection forms; data extracted from included studies; data used for all analyses; analytic code; any other materials used in the review. | Full supplmentary data is available for the authors under request |
